# Ischemic Stroke and the Biological Hallmarks of Aging

**DOI:** 10.14336/AD.2024.1059

**Published:** 2025-09-30

**Authors:** Fangyuan Cheng, Bo Yan, Pan Liao, Han Gao, Zhenyu Yin, Dai Li, Ping Lei

**Affiliations:** ^1^Department of Geriatrics, Tianjin Medical University General Hospital, Tianjin, China.; ^2^Key Laboratory of Post-Trauma Neuro-Repair and Regeneration in Central Nervous System, Tianjin Key Laboratory of Injuries, Variations and Regeneration of Nervous System, Tianjin Neurological Institute, Ministry of Education, Tianjin, China.; ^3^Haihe Laboratory of Cell Ecosystem, Department of Geriatrics, Tianjin Medical University General Hospital, Tianjin, China.; ^4^School of Medicine, Nankai University, Tianjin, China.

**Keywords:** aging, ischemic stroke, hallmarkers, biology

## Abstract

With the advent of an aging population, the study of aging and related research has been increasingly prominent, focusing on how to fully understand and delay aging—a key concern for contemporary medical professionals. Stroke is an acute focal neurological deficit. Globally, ischemic stroke accounts for only 60-70% of all strokes, meanwhile, it is the second leading cause of death. With the introduction of the concept of biomarkers of ageing, the research of ischemic stroke or acute brain injury in relation to these biomarkers has remained fragmented. In this review, we aim to consolidate the current evidence, highlighting the intricate relationship between ischemic stroke and aging-related hallmarks during its occurrence. By providing a comprehensive overview, we hope to offer researchers a broader perspective on how acute injury mechanisms intertwine with aging. We hope to present a new viewpoint and a more complete evaluation framework for future research and exploration in the field of aging.

## 1. Introduction

Stroke is an acute focal neurological deficit, with no more prominent explanation other than cerebrovascular causes [1]. Common symptoms include hemiplegia, dysarthria, sensory disturbances, aphasia, and visual impairment [2]. Globally, ischemic stroke (IS) accounts for only 60-70% of all strokes [3]. In some high-income countries, stroke affects up to one in five individuals over their lifetime, while in low-income countries, the impact rate is nearly one in two [4]. Worldwide, it ranks as the second most common cause of mortality [4, 5].

**Figure 1.**
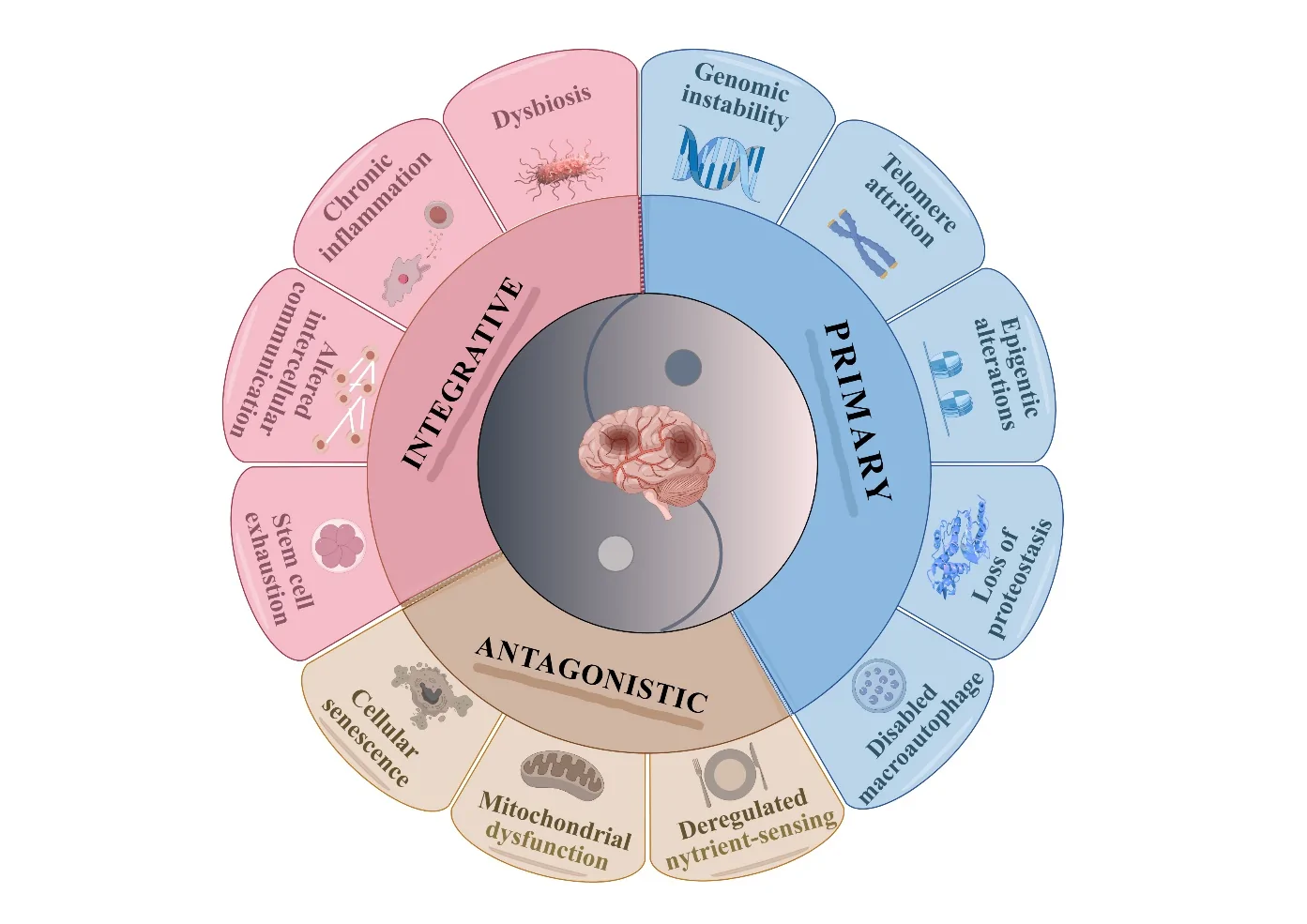
**Ischemic stroke and the hallmarks of aging**. The framework consolidates the twelve hallmarks of aging identified in this study: genomic instability, telomere attrition, epigenetic changes, loss of proteostasis, impaired macroautophagy, deregulated nutrient sensing, mitochondrial dysfunction, cellular senescence, stem cell depletion, altered intercellular communication, chronic inflammation, and dysbiosis. These hallmarks are categorized into three distinct groups: primary, antagonistic, and integrative. 12 biomarkers are related to each other and to ischemic stroke like Tai Chi. These biomarkers have their own individual effects on ischemic stroke, yet they are inextricably linked and intricately intertwined.

With the advent of an aging population, the study of aging and related research has been increasingly prominent, focusing on how to fully understand and delay aging—a key concern for contemporary medical professionals. Since the first edition of “The Hallmarks of Aging” was published in *Cell* in 2013, a vast number of related research articles have been published [6]. In 2022, Carlos López-Otín and colleagues updated this content, providing a deeper understanding of the concept of aging [7]. In this updated article, the authors expanded the original nine molecular, cellular, and systemic hallmarks of aging to twelve, which now include: genomic instability, telomere attrition, epigenetic alterations, loss of proteostasis, disabled macroautophagy, deregulated nutrient-sensing, mitochondrial dysfunction, cellular senescence, stem cell exhaustion, altered intercellular communication, chronic inflammation, and dysbiosis (Fig.1).

Recently, our team published an article in the *Immunity* on ischemic stroke and atherosclerosis. We discovered that cerebral ischemia induces sustained activation of peripheral endothelial Notch1 signaling, leading to the generation of senescent pro-inflammatory endothelial cells. This, in turn, accelerates myeloid cell adhesion and the progression of atherosclerosis [8]. Although numerous studies have explored aging-related phenotypes in the context of stroke, a comprehensive review systematically summarizing the connections between ischemic stroke and aging-related hallmarks is still lacking. In this review, we aim to consolidate the current evidence, highlighting the intricate relationship between ischemic stroke and aging-related hallmarks during its occurrence. This understanding may provide new insights into the pathological mechanisms of ischemic stroke and inform future research on both ischemic stroke and aging.

## 2. Pathophysiology of IS

Ischemic stroke is among the foremost contributors to mortality and morbidity globally, characterized by the abrupt onset of neurological deficits due to a substantial decrease or complete interruption of cerebral perfusion [9]. Principal pathological features of ischemic stroke include extensive neuronal apoptosis, disruption of axonal integrity, production of reactive oxygen species and free radicals, cerebral edema, dysfunction of the blood-brain barrier (BBB), and resultant neuroinflammation, ultimately leading to local neural tissue and functional loss [10, 11]. The primary objective of clinical intervention in stroke is the rapid restoration of regional cerebral perfusion to mitigate the extent and severity of neurological impairment [12]. However, irreversible injury to neural tissue frequently occurs due to the restricted therapeutic window and secondary brain injury mechanisms [13]. Moreover, post-reperfusion, the surviving neural cells typically exhibit limited capacity for regeneration and replacement of necrotic functional neural tissue.

## 3. Cellular Senescence and IS

**Figure 2.**
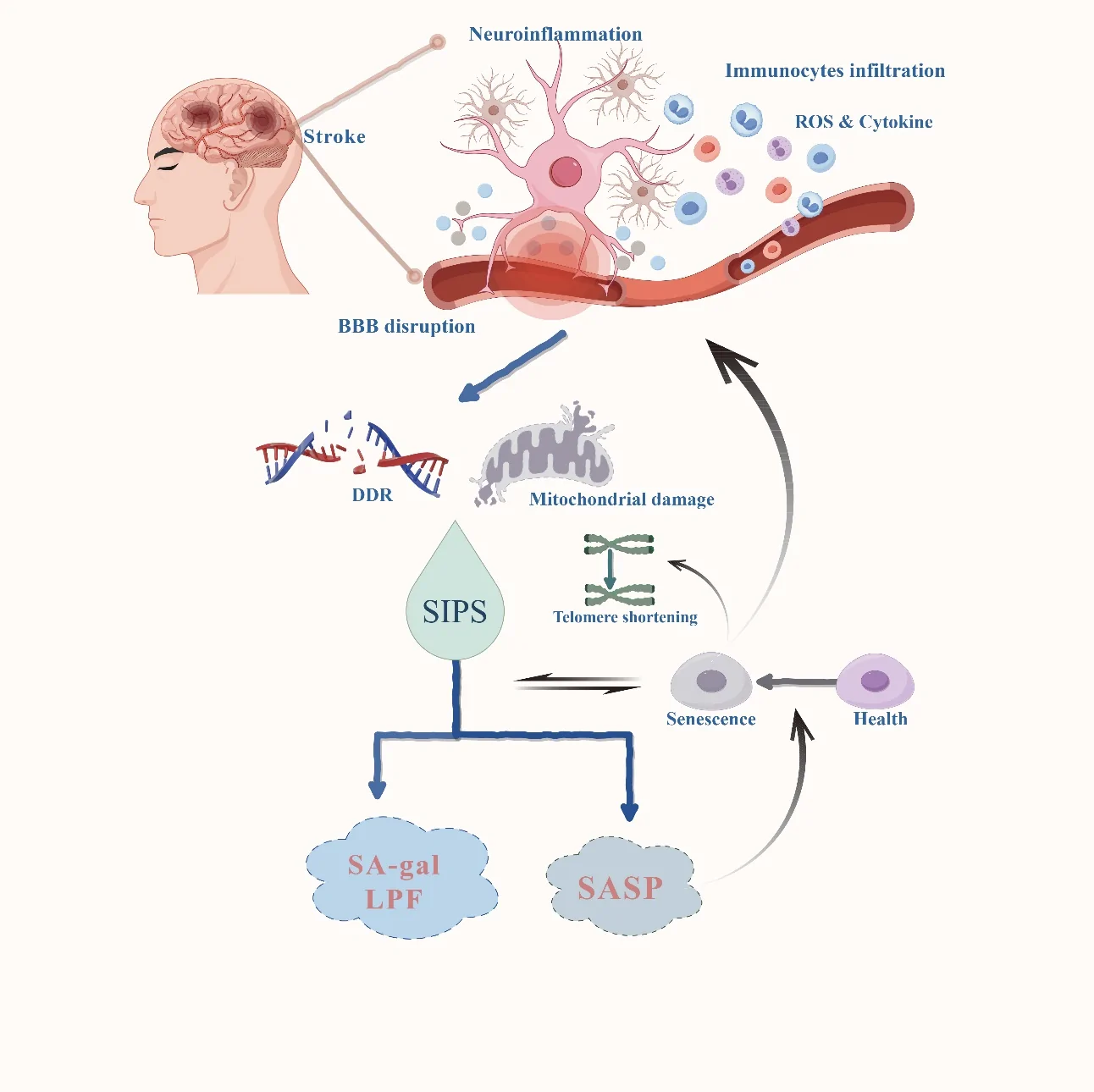
**Cellular senescence, neuroinflammation, SASP and Ischemic stroke**. Ischemic stroke contributes to the disruption of the blood-brain barrier and triggers an intense neuroinflammatory response. The neuroinflammation in ischemic brain tissue is intensified with the infiltration of immune cells and the release of ROS and multiple pro-inflammatory cytokines. These pathological reactions cause DNA damage response and mitochondrial damage, which not only contribute to the progression of inflammation, but also cause SIPS (stress-induced premature senescence) in brain tissue cells. SIPS induces the development of the senescence-associated secretory phenotype, SASP, and the deposition of senescence-associated markers, SA-gal and lipofuscin, which are pathological alterations that contribute to the genesis of senescent cells. The ongoing occurrence of senescent cells and continued secretion of SASP also exacerbate neuroinflammation in brain tissue. This makes the damage of ischemic stroke no more just an effect caused by acute injury, but also has a non-negligible role in the senescence of brain cells.

Cellular senescence is defined by a permanent cessation of proliferation that ensues when cells encounter replicative exhaustion, oncogenic insult, cellular stress, or genomic instability [14]. Senescent cells share common morphological and functional characteristics, such as enlarged cell size, accumulation of senescence-associated β-galactosidase, and the presence of lipofuscin in the cytoplasm [15-17]. Factors inducing primary senescence encompass oncogenic signaling, genotoxic insults, critically shortened telomeres, mitochondrial dysfunction, infections (viral or bacterial), oxidative stress, nutrient imbalances, and mechanical stress [18]. Additionally, secondary or paracrine senescence may be initiated by an inflamed and fibrotic extracellular matrix [19]. Current studies highlight several key characteristics of cellular senescence, including the presence of DNA damage foci, condensed regions of heterochromatin, telomere shortening, and increased expression of cell cycle regulators such as p16 and p21 [15-17]. Additional significant characteristics encompass the inability to trigger pro-apoptotic pathways, dependence on immune cells for tissue clearance, and alterations in the secretome, referred to as the senescence-associated secretory phenotype (SASP) [20, 21](Figure.2).

Certain investigations indicate that the upregulation of p16 and p21 mRNA is confined to the infarcted areas of the brain in transient middle cerebral artery occlusion (tMCAO) models. Furthermore, the expression of pro-inflammatory cytokines such as IL-6, TNF-α, and CXCL1, along with their receptor CXCR2 mRNA, shows a strong correlation with p16/p21 mRNA levels. p16 is primarily expressed in the neuronal cytoplasm and the cytoplasm/membrane of microglia. p21 is observed in the neuronal cell membrane and exhibits a mixed cytoplasmic and membrane pattern in microglia as well [22].Similarly, another study indicated that in the tMCAO animal model, lipofuscin accumulation in lysosomes is evident from the onset of brain injury and continues to increase until it is abundantly distributed in the injured brain regions by 7 days post-ischemia. Lipofuscin granules are localized within infarcted tissue, impacting both the cortex and striatum. In the ischemic hemisphere, levels of cell cycle arrest markers such as p21, p53, and p16INK4a, along with pro-inflammatory cytokines IL-6, TNF-α, and IL-1β, are markedly elevated compared to their non-ischemic contralateral counterparts [23].

Serving as a vital marker of cellular senescence, telomeres are nucleoprotein structures found at chromosome ends, consisting of TTAGGG repeat sequences and a 3’-rich single-stranded overhang [24]. They are essential for maintaining DNA stability and integrity. Telomeres shorten with each cell division, and upon reaching a critical threshold, they signal the cell to cease further proliferation and enter a state of senescence, known as replicative senescence [25]. A prospective clinical cohort study not only confirmed the association between ischemic stroke and shorter telomere length but also suggested that telomere length may be particularly relevant to stroke phenotype in the acute stroke setting [26]. Other studies conducted on Chinese populations have indicated that shorter telomere length is associated with ischemic stroke, serving as a strong predictor of post-stroke mortality and is independently linked to thrombotic stroke and lacunar infarction [27, 28].

## 4. Altered intercellular communication and IS

Intercellular communication is a broad concept that includes almost all recognized physiological functions. It is typically defined as the transfer of information between cells via paracrine, autocrine, endocrine, or direct cell-to-cell signaling [29]. Since IS is an acute injury, the processes and pathological changes in intercellular communication are more complex compared to other diseases. Therefore, in this review, we will discuss intercellular communication from the perspectives of inflammation and the senescence-associated aecretory phenotype (SASP) (Fig. 2).

## 4.1 Inflammation

Extensive research has demonstrated that acute inflammation in ischemic stroke (IS) manifests immediately after vascular occlusion. Alongside the activation of microglia in the ischemic brain, the infiltration of circulating cells—including granulocytes, neutrophils, monocytes/macrophages, and T cells—further intensifies cellular death. These cells have been consistently identified in peripheral blood samples from patients with ischemic stroke [30-34]. In the acute phase, lasting from minutes to hours, the injured tissue emits reactive oxygen species (ROS) and pro-inflammatory mediators, including chemokines and cytokines, which facilitate leukocyte adhesion and transendothelial migration [35].

Within 24 hours of onset, cellular damage, ATP depletion, fibrinogen, and reactive oxygen species (ROS) activate microglia, which are the primary immune cells of the central nervous system (CNS) [36, 37]. Microglia secrete inflammatory cytokines (such as IL-1β, IL-6, and TNF-α) and chemokines (such as MIP-1, MIP-2, and MCP-1) within the brain parenchyma [38], promoting NF-κB expression and enhancing the upregulation of endothelial cell surface adhesion molecules (E-selectin, L-selectin, P-selectin, ICAM-1, and integrins) [39]. The release of cytokines also promotes progressive cell death [40] and oxidative cellular damage [41], exacerbating the ischemic cascade.

In the subacute phase following the acute stage, infiltrating leukocytes continue to release cytokines and chemokines, and importantly, they release excessive reactive ROS [42]. This, in turn, promotes MMPs (the production of matrix metalloproteinases), particularly MMP-9, and NO (nitric oxide) [43, 44]. Jalal et al. and multiple research teams have shown that the activation of MMPs intensifies the inflammatory response, resulting in disruption of the blood-brain barrier, cerebral edema, neuronal death, and hemorrhagic transformation, which is driven by excessive protease activation and resident immune cells, further enhancing leukocyte infiltration [45, 46].

Studies using rodent models have shown that, in the months following a stroke, inflammation can spread from the peri-lesional region to secondary areas of heightened inflammatory response across the entire brain [47]. Even one-month post-stroke, activated microglial populations in these regions continue to exhibit inflammatory phagocytic activity. Research has indicated that in mouse models, microglial activity and the presence of CD4^+^T cells, CD8^+^T cells and B cells can still be observed in the infarct area 30 days after ischemia, with a notable accumulation of foam cells containing lipid droplets [48, 49].

Increased production of TNF-α is thought to play a role in neuronal loss through the enhanced phagocytic activity of microglia [50]. Increased levels of TNF-α from activated microglia have been demonstrated to persist for a minimum of 15 months following stroke in the human brain [51]. Additionally, at 84 days post-stroke, microglia represent the most transcriptionally active immune cells in mice [52].

## 4.2 Senescence-associated secretory phenotype (SASP)

Cellular senescence represents a stress response resulting in irreversible cell cycle arrest and significant phenotypic alterations, including the secretion of a bioactive profile referred to as the SASP (Senescence-associated Secretory Phenotype) [53]. SASP occurs in all senescent cells and is produced via multiple pathways such as cellular injury, DNA damage, oncogenic transformation, inflammation, and oxidative stress. It establishes a pro-inflammatory phenotype by secreting detrimental cytokines and additional markers into the adjacent cellular environment through paracrine signaling [22, 54-56]. Due to the diverse functions of SASP, research into its role in ischemic stroke has become increasingly prominent as studies continue to advance.

Stress-induced premature senescence (SIPS) mediated by ischemic injury is an exceptionally complex process that remains incompletely understood [57]. Ischemia-reperfusion (I/R) injury induces ROS, leading to oxidative stress and mitochondrial damage [58]. The elevation of ROS and mitochondrial injury is regarded as the DAMP (damage-associated molecular pattern), leading to neutrophil extravasation and the overproduction of pro-inflammatory cytokines, consequently resulting in SIPS [59]. Senescent cells further propagate inflammation by secreting SASP, which induces senescence in adjacent cells [60]. I/R injury triggers sustained DNA damage responses and induces cell cycle arrest through the activation of p53/p21 and p16/pRb signaling pathways [14, 61].

Interestingly, many stressors that trigger senescence play an active role in the pathophysiology of IS. Lately, there has been increasing recognition of the relationship between cellular senescence, SASP and brain injury and neurodegeneration [49]. Experimental findings demonstrate that ischemic stroke initiates SASP, with the release of cytokines including p16, p21, IL-6, TNF-α, CXCL1, and CDK4, along with the chemokine receptor CXCR-2 mRNA in the ipsilateral penumbra 30 minutes following MCAO, showing pronounced activation 72 hours post-MCAO [22]. The predominant localization of these markers occurs in neurons and microglia, with their presence in membrane and cytoplasmic compartments signaling a DDR (DNA damage response) [62]. Enhanced SASP activation is linked to the activation of the p65/NF-κB pro-inflammatory pathway in microglia and an increased expression of DNA repair foci (H2AX). NF-κB serves as a pivotal regulator of SASP transcriptional activation [63]. Cellular senescence is first detected in the penumbra surrounding the injury and subsequently in the ischemic core, where increased p21 levels potentially protecting neurons by preventing cell cycle progression.

SA-β-gal (senescence-associated β-galactosidase) serves as a biomarker for cellular senescence [64]. At 72 hours post-MCAO, infarcted brain regions lack SA-β-gal activity, indicating that senescence occurs later. Immunohistochemical analysis of postmortem human brain samples shows elevated levels of p16 in the periphery of infarcted areas [22]. Similarly, in the MCAO mouse model, the mRNA levels of p16, IL-6, Ccl8, and Cxcl2 were elevated in the ipsilateral cortex, striatum, and hippocampus between 6 and 12 hours post-ischemia [65]. Immunohistochemical analysis reveals that the heightened levels of senescence markers are mainly concentrated in astrocytes and endothelial cells, as opposed to neurons and microglia within the ipsilateral area. Compared to young mice, aged mice demonstrate an increased proportion of senescent neurons, microglia, astrocytes, and endothelial cells, suggesting that brain lesions resulting from acute senescence closely parallel those found in aged brains. Cells progress to senescence in response to diverse cellular injuries, distinguished by enhanced resistance to apoptosis, cell cycle arrest, and elevated levels of SASP [66]. In a rat model of IS, significant evidence of cellular senescence appears 7 days after the insult, characterized by lipofuscin accumulation in the cortex and caudate-putamen regions, elevated levels of mediators associated with cell cycle arrest (including p21, p53, and p16), and increased SASP secretion [67]. Lipofuscin constitutes an indigestible lysosomal complex formed from lipids, proteins, ions, and sugars, notably within post-mitotic cells [68]. Its accumulation in lysosomes of post-mitotic senescent cells makes it a lysosomal marker of senescence, alongside SA-β-gal [69]. Elevated lipofuscin accumulation may be observed as soon as 24 hours after MCAO, indicating a potentially targetable pathogenic event. This contrasts with earlier findings reported by Torres-Querol et al. [22], Baixauli-Martin et al. propose that lipofuscin serves as a more reliable marker of senescence than SA-β-gal [67]. An extensive computational analysis of RNA-seq data from public repositories indicates variations in the expression of senescence-related genes (such as ANGPTL4, Ccl3, Ccl7, Cxcl16, and Tnf) following a stroke, highlighting species conservation [70]. In summary, these investigations highlight the role of cellular senescence and SASP in the pathophysiology of IS, indicating their promise as avenues for therapeutic intervention.

Existing studies examining the effects of senescence modulation and SASP on functional outcomes in IS are still sparse. Studies indicate that the removal of senescent cells from the brain via genetic and pharmacological strategies positively impacts cognitive functions associated with aging [71]. In light of these results, we believe that further investigation into the regulation of SASP and cellular senescence is crucial for mitigating stroke outcomes and for a better understanding of the intricate relationship between SASP, cellular communication, and ischemic stroke.

## 5. Mitochondrial dysfunction and IS

**Figure 3.**
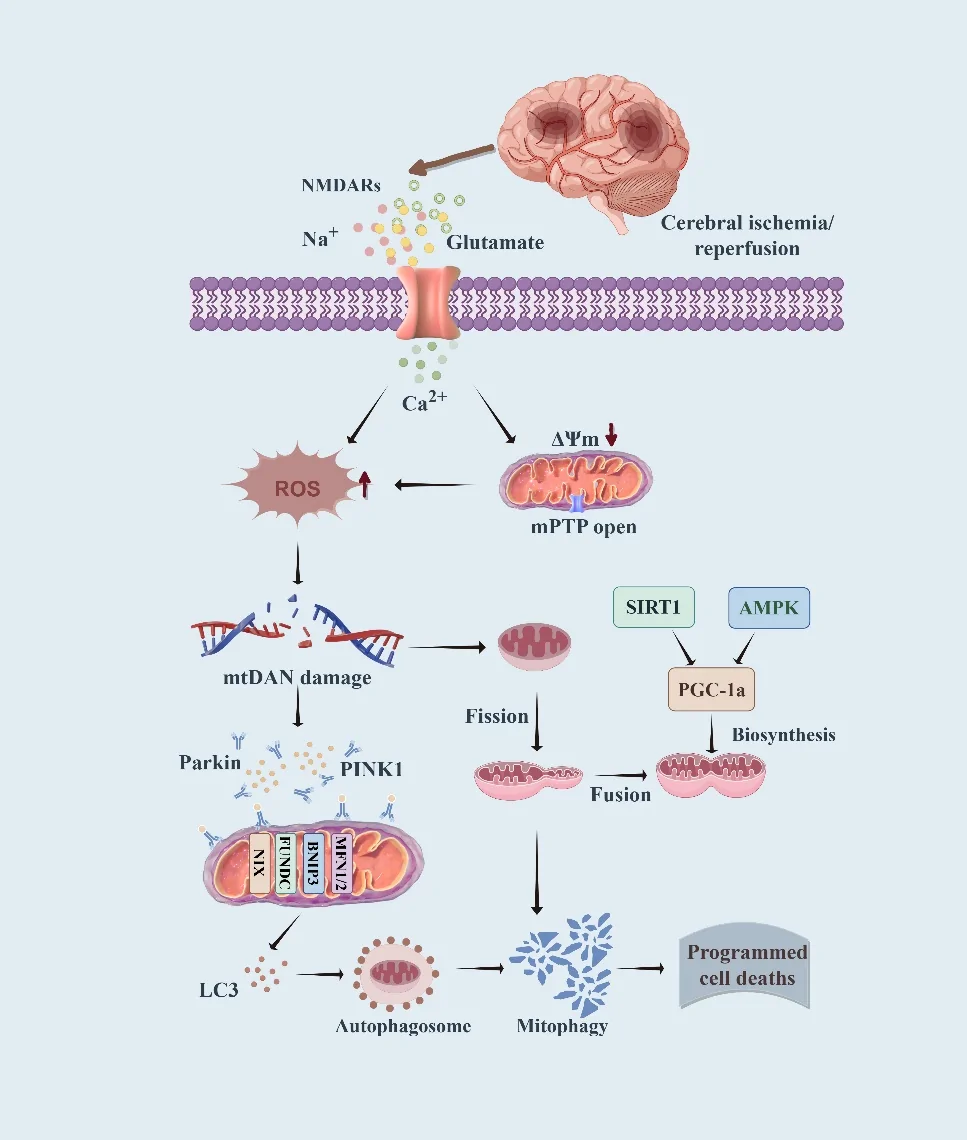
**Mitochondrial dysfunction and Ischemic stroke**. Mitochondria ranks among the organelles most vulnerable to ischemic injury in the brain. ROS bursts, Ca^2+^overload, excitotoxicity, and other consequences of ischemia/reperfusion (I/R) can lead to mitochondrial abnormalities and dysfunction. During brain ischemia, there is a dramatic reduction in energy supply. The inner mitochondrial membrane (IMM) and mitochondrial cristae structures undergo deformation due to oxidative stress and Ca^2+^ overload, triggering mitochondrial responses. However, in response to ischemic and hypoxic stress, mitochondrial oxidative stress induced by ROS can disrupt the balance between mitochondrial fusion and fission. This disruption results in increased mitochondrial fragmentation and fission as well as imbalance of mitochondrial autophagy, which heightens neuronal susceptibility to cell death.

Mitochondria function as the cellular energy hub and are also potential initiators of inflammation (via the activation of inflammasomes or cytosolic DNA sensors upon the leakage of reactive oxygen species or mitochondrial DNA) and cell death (upon the release of caspase activators, nucleases, or other cytotoxic enzymes from the intermembrane space) [72]. With advancing age, mitochondrial functionality declines as a result of various interconnected mechanisms, such as the buildup of mtDNA mutations, disturbances in protein homeostasis that destabilize respiratory chain complexes, diminished organelle turnover, and modifications in mitochondrial dynamics [73]. This scenario undermines the role of mitochondria in cellular bioenergetics, elevates ROS generation, and could precipitate unintentional permeabilization of the mitochondrial membrane, resulting in inflammation and cell death [74] (Fig. 3).

### 5.1 Mitochondrial Abnormalities and Dysfunction in IS

Mitochondria rank among the organelles most vulnerable to ischemic injury in the brain [75]. Bursts of reactive oxygen species (ROS), calcium overload, excitotoxicity, and additional effects of ischemia/reperfusion (I/R) can result in mitochondrial abnormalities and dysfunction [76]. During brain ischemia, there is a dramatic reduction in energy supply. The IMM (inner mitochondrial membrane) and mitochondrial cristae structures undergo deformation due to oxidative stress and Ca^2+^ overload, triggering mitochondrial responses such as excessive ROS production, mitochondrial Ca^2+^ overload, and disruption of mitochondrial quality control (MQC) mechanisms [77].

Mitochondrial oxidative stress refers to a condition arising from the imbalance between oxidation and antioxidation within the mitochondrial respiratory chain [78], playing a crucial role in ischemia/reperfusion (I/R) events [79]. Following brain ischemia, reduced oxygen levels in mitochondria limit mitochondrial oxidative phosphorylation(OXPHOS), decreasing ATP production and resulting in the release of ROS and incomplete metabolites, such as O^2-^ (superoxide anions), OH- (hydroxyl radicals), RNS (reactive nitrogen species), and NO from the ETC (electron transport chain) [80, 81]. During reperfusion, upon restoration of oxygen supply, pro-oxidant enzyme systems and mitochondria utilize oxygen to generate ROS, leading to a temporary yet pronounced ROS surge that sets off a cascade of reactions, impacting cell signaling pathways and potentially resulting in cell death [82]. Additionally, the process of reperfusion markedly diminishes the activity of succinate dehydrogenase and cytochrome c oxidase, alongside other critical enzymes in the electron transport chain, resulting in reduced efficiency of oxidative phosphorylation and compromised ATP synthesis [83]. During reperfusion, Ca^2+^ influx and ROS bursts contribute to mitochondrial swelling, increased cytosolic density, depolarization of the mitochondrial membrane potential (ΔΨm), and the opening of the MPTP (mitochondrial permeability transition pore) [77].

Following I/R, mitochondria initiate various pathways for the repair and clearance of mtDNA, including direct reversal (DR), mismatch repair (MMR), base excision repair (BER), double-strand break (DSB) repair, and other mechanisms for mtDNA repair [84]. Within mitochondria, BER is the predominant pathway for rectifying different forms of DNA damage that impact the nuclear genome [85]. When these repair pathways fail to adequately restore mtDNA integrity and functionality, irreversible alterations arise, resulting in mtDNA mutations [86]. Moreover, mtDNA mutations alter tRNA structures, impairing the assembly and enzymatic activity of respiratory chain complexes, further amplifying ROS generation and worsening mtDNA mutations, thereby establishing a detrimental cycle in I/R. Research indicates that I/R can cause mtDNA damage. While mtDNA can self-repair after brief cerebral ischemia (<30 min), damage after prolonged cerebral ischemia is irreversible, diminishing the function of Complexes I and IV within the electron transport chain (ETC) and compromising the integrity of electron transfer [87]. Mitochondrial DNA mutations also influence mitochondrial autophagy. In comparison to healthy cells, mtDNA mutant cells display decreased levels of the autophagy marker protein light chain 3 (LC3) and an increased accumulation of the autophagy substrate p62, resulting in impaired mitochondrial autophagy and a marked rise in ROS levels [88].

## 5.2 Mitochondrial Fission and Fusion in IS

Mitochondria are highly dynamic organelles that regularly participate in fission and fusion processes, essential for preserving mitochondrial integrity and bioenergetics, as well as for sustaining cellular homeostasis in both physiological and pathological conditions [89]. In a healthy state, mitochondria perpetually modify their morphology, dimensions, and quantity via fusion and fission processes to accommodate cellular metabolic requirements [89]. However, in response to ischemic and hypoxic stress, mitochondrial oxidative stress resulting from ROS may disturb the equilibrium between mitochondrial fusion and fission. This disruption results in increased mitochondrial fragmentation and fission, which heightens neuronal susceptibility to cell death [90].

In the context of I/R, increased ROS concentrations compromise mitochondrial membrane potential and induce depolarization. This disruption prompts Drp1 to localize to the OMM (outer mitochondrial membrane) by interacting with Fis1, the fission factor (MFF), and proteins associated with mitochondrial dynamics (MiD49/MiD51) [91]. Studies conducted both in vitro and in vivo have demonstrated that Drp1 and phosphorylated Drp1 (P-Drp1) levels are upregulated after tMCAO, indicating increased mitochondrial fission under I/R conditions. Inhibition of Drp1 or the use of siRNA targeting Drp1 has been shown to mitigate mitochondrial fission and has beneficial effects on cerebral ischemia [92, 93].Under hypoxic conditions, I/R may hinder mitochondrial fusion by decreasing OPA1 or reducing levels of MFN2, thereby disrupting intracellular homeostasis and triggering neuronal death [94, 95]. Additionally, the downregulation of MFN2 exacerbates I/R damage by inhibiting autophagosome formation and preventing the fusion of autophagosomes with lysosomes [94].

Mitochondrial autophagy, a selective form of autophagy, refers to the process of specifically eliminating damaged or dysfunctional mitochondria to prevent excessive ROS production and subsequent cell death [96]. Among the various mechanisms of mitochondrial autophagy in mammalians, the PINK1/Parkin pathway has been among the most thoroughly investigated.

Under physiological conditions, autophagy facilitates the clearance of misaggregated proteins and damaged organelles within cells. However, excessive autophagy can lead to excessive and unnecessary cell death [97]. Following I/R, PINK1 builds up on the OMM, triggering the relocation of Parkin to the impaired brain region and raising the concentrations of other autophagy-related proteins like LC3 and Beclin1 [98]. Researchers have found that stimulating mitochondrial autophagy via the PINK1/Parkin axis may decrease the aggregation of impaired mitochondria and alleviate neuronal injury during I/R [99-101]. Additionally, literature indicates that the degradation of NIX leads to a lack of mitochondrial autophagy in ischemic brain conditions [102]. Overexpression of FUNDC1 can inhibit cell apoptosis and enhance mitochondrial function against I/R damage [103].

## 6. Deregulation of nutrient sensing and IS

The nutrient-sensing system, which has been conserved across evolution, comprises extracellular ligands such as insulin and IGF (insulin-like growth factors) that interact with receptor tyrosine kinases and engage intracellular signaling pathways [104]. These signaling cascades include the PI3K-AKT and Ras-MEK-ERK pathways, in addition to transcription factors like FOXO and E26 that promote the activation of genes implicated in a range of cellular functions [7]. The MTORC1 (mechanistic target of rapamycin complex 1) reacts to various nutrients, such as glucose and amino acids, as well as stress factors like hypoxia and low energy, thereby influencing the functionality of multiple proteins, including transcription factors like SREBP and TFEB [7]. This network functions as a crucial regulator of various cellular processes, such as autophagy, the biogenesis of protein synthesis, mRNA and ribosomes, and metabolism of glucose, nucleotides, and lipids, in addition to mitochondrial biogenesis and proteasome function [7]. In the presence of nutrients and low stress, the network adapts by stimulating anabolic processes or activating cellular defense mechanisms in response to stress and nutrient scarcity [105].

## 6.1 IGF-1 in IS

The neuroprotective effects of IGF primarily operate through mechanisms similar to those of insulin. In vitro studies have demonstrated that insulin can prevent excitotoxicity and oxidative stress [106-108], and it possesses strong anti-apoptotic properties [109]. In vivo research has confirmed its protective effects against hypoxic-ischemic injury [110], with insulin often considered a factor that promotes neuronal survival [111]. In addition to its role in mitigating acute damage, IGF-I is vital for long-term recovery after ischemic stroke, as it promotes the proliferation and differentiation of oligodendrocyte progenitor cells [112] and enhances myelin synthesis in vitro [113], both of which are essential for recovery of the brain post-stroke [114, 115]. Emerging clinical research indicates a negative correlation between plasma IGF-1 concentrations and the risk of ischemic stroke [116-118]. Furthermore, lower circulating IGF-1 concentrations have been linked to unfavorable outcomes following stroke [119, 120].

Numerous animal studies have investigated the effects of IGF-1 treatment in experimental models of AIS (acute ischemic stroke) induced by MCAO employing diverse intervention strategies. For instance, subcutaneous administration of IGF-1 at 200 μg/d for 7 days, starting 30 minutes post-injury, reduced final infarct volume and significantly improved functional outcomes [121]. Another study demonstrated that acute IGF-1 administration in diabetic rats, either 30 minutes prior to or 2 hours subsequent to MCAO, followed by 24 hours of reperfusion, resulted in a decreased lesion volume as assessed by MRI and a reduced count of apoptotic cells in the cortical penumbra [122]. The administration of an adeno-associated IGF-1 construct for gene transfer was assessed in Sendai virus-infected mice, revealing neuroprotective effects and enhanced survival rates when delivered 30 minutes post bilateral artery occlusion [123]. Direct intraventricular injection of IGF-1, administered 30 minutes after stroke induction, was found to reduce infarct size and improve functional outcomes [121], while local application of IGF-1 to the cerebral cortex achieved similar effects in reducing infarct size and enhancing neuron survival [124]. Additionally, the effectiveness of intranasal IGF-1 delivery was investigated in animal MCAO models, revealing time-dependent beneficial effects, with reduced apoptosis in the infarct zone and significant improvements in motor and sensory functions in rats [125, 126].

## 6.2 AMPK and mTORC in IS

In brain tissue, low glucose levels can reduce ATP levels in glial cells and neurons, increasing ROS, thereby activating AMPK and encouraging metabolic reprogramming [127]. In cellular models, deprivation of glucose or administration of the hexokinase inhibitor 2DG (2-deoxyglucose) leads to a reduction in glycolytic flux and ATP, resulting in the inhibition of mTORC1. In states of energy deficiency, AMPK phosphorylates TSC2, thus enhancing its function [128, 129]. The activation of TSC1/2 results in the inactivation of Rheb and the phosphorylation of other substrates [130, 131], thereby suppressing mTORC1 activity [128, 132, 133]. Consequently, the mTORC1 complex becomes inactive. Additionally, AMPK activation may enhance the modulation of cellular energy levels through diverse mechanisms, such as directly phosphorylating glucose transporter GLUT4 [134] or influencing mitochondrial biogenesis via Sirt1 or PGC-1 [135], which subsequently affects energy production in ischemic cells or tissues.

A significant aspect during IS is the temporary or permanent reduction in oxygen availability. Likewise, this pathological state diminishes cellular ATP levels by suppressing metabolic pathways [127]. Similar to low glucose levels, AMPK activation inhibits mTORC1 through the phosphorylation of TSC2 and Raptor. Disruption of the mTORC1 complex parallels the effects of pharmacological mTOR inhibitors such as rapamycin, resulting in diminished mitochondrial membrane potential, reduced oxygen consumption, and lower ATP synthesis capacity [136].

Additionally, several regulatory factors have been identified that can inhibit mTORC1. Following hypoxia/ischemia, the Regulated in Development and DNA Damage Response 1 (REDD1; also known as RTP801/DDIT4) protein suppresses mTORC1. Initially characterized in Drosophila, this gene encodes a hypoxia-inducible transcript that inhibits mTORC1 [137, 138]. REDD1, a conserved protein involved in stress response, modulates mTORC1 [139] and is thought to inhibit mTORC1 by regulating the dissociation of TSC2 from its adaptor protein 14-3-3, which in turn stabilizes the association between TSC1 and TSC2 [140, 141]. Likewise, the promyelocytic leukemia (PML) tumor suppressor protein is known to interact with mTOR, rendering it inactive under hypoxic conditions by sequestering it within nuclear bodies [142, 143].

## 7. Epigenetic alterations and IS

**Figure 4.**
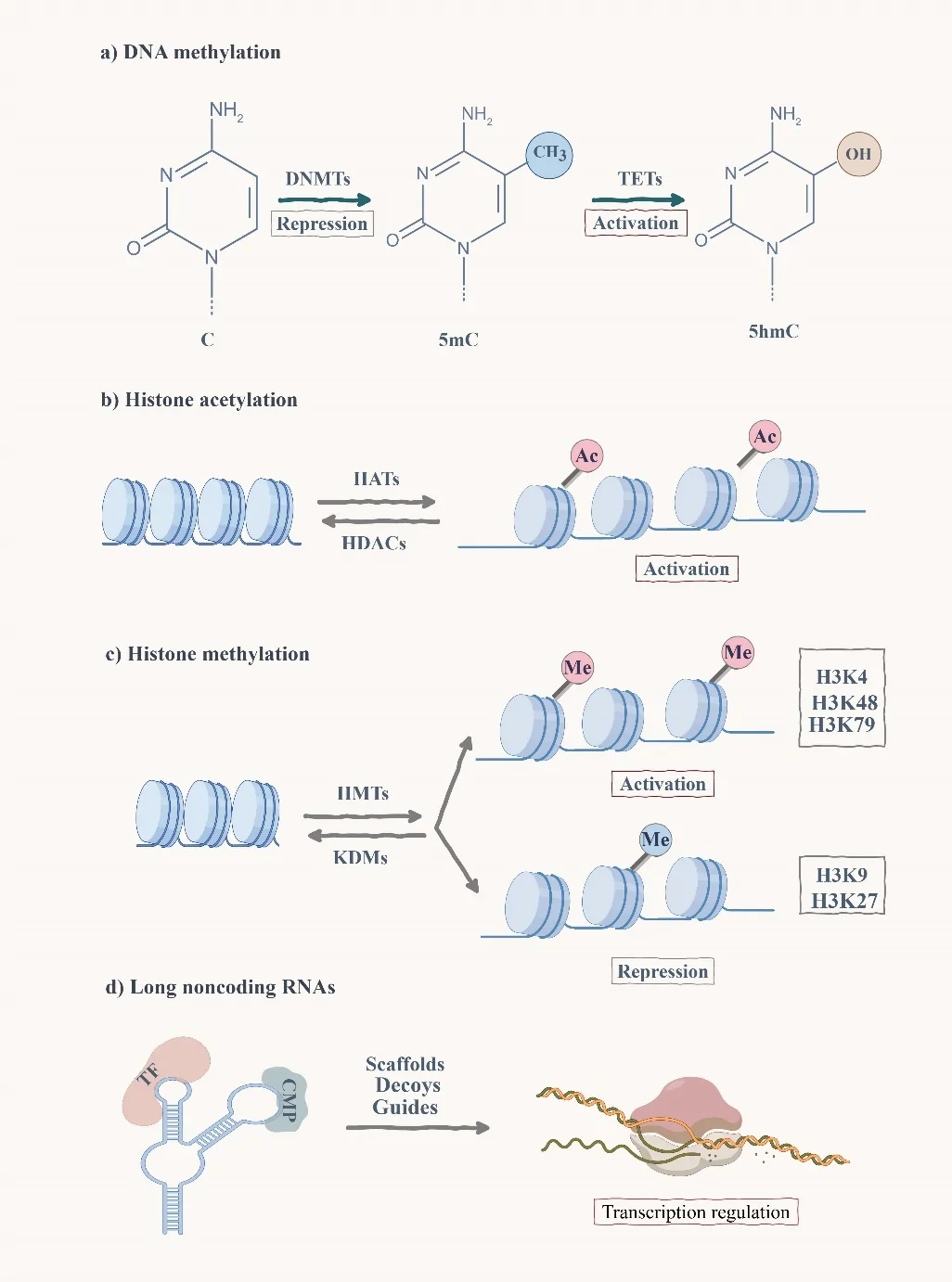
**Epigenetic control of gene expression**. Chemical modifications of DNA and histones, along with noncoding RNAs, epigenetically regulate gene expression. (**A**) DNA methylation is mediated by DNA methyltransferases (DNMTs), which add a methyl group to cytosine to produce 5-methylcytosine (5mC), subsequently converted to 5-hydroxymethylcytosine (5hmC) by ten-eleven translocation (TETs) dioxygenases. 5mC serves as a repressive mark, while 5hmC functions as an activating mark for gene expression. (**B**) Histone acetyltransferases (HATs) facilitate the transfer of acetyl groups to lysine residues, while histone deacetylases reverse this modification. Histone acetylation relaxes chromatin structure and promotes gene expression. (**C**) Histone methyltransferases (HMTs) facilitate the addition of methyl groups to lysine or arginine residues, while histone demethylases (KDMs) reverse this modification. The position and extent of methylation determine its function in gene regulation. For instance, trimethylation of histone 3 at lysine residue 4 (H3K4me3) activates transcription, while H3K27me3 represses transcription. (**D**) Noncoding RNAs interact with regulatory proteins in the nucleus, such as chromatin-modifying proteins and transcription factors, serving as scaffolds, decoys, or guides to regulate gene expression.

Epigenetics encompasses heritable and modifiable mechanisms that influence gene expression without modifying the DNA sequence itself [144]. The regulation of epigenetics is complex and is subject to influence from environmental factors, including stress, nutrition, and aging, which alter physiological conditions [145]. Various epigenetic modifications are critical during brain development and are vital for sustaining cerebral function throughout life, such as DNA methylation, hydroxymethylation, histone modifications, and long non-coding RNAs (lncRNAs) [146]. Additionally, epigenetics covers multiple dimensions of central nervous system pathology and is linked to the severity and progression of numerous neurological disorders[145]. A growing body of evidence suggests that epigenetic mechanisms modulate cerebrovascular pathology in both human and experimental models of stroke (Fig. 4).

## 7.1 DNA methylation in IS

DNA methylation predominantly occurs in CpG islands, which are concentrated regions of CG dinucleotide repeats found near gene promoter areas, and it plays a crucial role in the stable silencing of gene expression [147]. Within the DNA methylation process, two crucial factors closely linked to this mechanism are DNA methyltransferases (DNMTs) and the ten-eleven translocation (TET) family (TET1-3) [148]. DNMTs transfer a methyl group from the metabolite S-adenosylmethionine (SAM) to cytosine residues in DNA, leading to the formation of 5-methylcytosine (5mC) and thereby promoting DNA methylation. In contrast, TETs introduce a hydroxyl group to 5mC, producing 5-hydroxymethylcytosine (5hmC), and can further oxidize 5hmC to yield 5-formylcytosine (5fC) and 5-carboxycytosine (5caC). These modifications are involved in DNA demethylation by facilitating the removal of thymine bases through thymine DNA glycosylase [149].

Research on ischemic stroke (IS) and DNA methylation has a substantial history. Early epigenetic studies indicated that ischemia in experimental animal models of MCAO and tMCAO reperfusion strongly induces high levels of methylation changes (elevated 5-methylcytosine [5mC] and DNA methyltransferases [DNMTs] in ischemic brain tissue), which are associated with exacerbated brain damage. Recent studies have not only demonstrated that inhibiting DNMTs can improve motor function after stroke, regardless of age [150-158], but have also identified specific DNA methylation changes at particular loci that are related to stroke injury. For example, elevated methylation levels have been detected in several genes, including rho-associated coiled-coil protein kinase 2 (ROCK2), peroxisome proliferator-activated receptor gamma (PPARγ), neurite outgrowth inhibitor protein-A (Nogo-A), metalloproteinases 2 (TIMP2), and Nogo receptor, ras homolog gene family member A (RhoA) [159, 160]. Alterations in 5mC and 5-hydroxymethylcytosine (5hmC) within mitochondrial DNA (mtDNA) have also been noted in experimental models [159, 160]. Increased expression of TET2 subtype in ischemic brain hemispheres correlates with elevated 5hmC in the BDNF promoter. Furthermore, TET3 knockout mice exhibit increased edema, larger infarct size, and motor deficits after stroke, suggesting that TET3 may confer endogenous protection in the context of ischemic stroke [150, 156]. Although increases in 5fC (5-formylcytosine) and 5caC (5-carboxycytosine) have also been detected in peri-infarct areas [151], their mechanisms of action remain unclear.

Multiple investigations involving patients with ischemic stroke have demonstrated strong links between blood DNA methylation and outcomes following the event. Notably, the methylation patterns in blood for the SLC6A4 (solute carrier family 6 member 4) gene correlate with the likelihood of stroke recurrence, post-stroke depression, and decline in neurological function [161-163]. Additionally, the methylation levels of various genes in the bloodstream are associated with stroke risk and functional outcomes in patients suffering from comorbidities like hypertension and obesity [164-166]. Moreover, analyses indicate that biological age, determined by circulating concentrations of 5mC(5-methylcytosine), may more accurately forecast stroke risk, outcomes, and recurrence than chronological age [167-171]. Collectively, these findings suggest that the concentration of methylated DNA in blood may act as important biomarkers for stroke diagnosis, prognosis, and therapeutic strategies.

## 7.2 Histone Modifications in IS

Histones are proteins responsible for packaging and structuring DNA into chromatin [151]. Various histone modifications have been characterized, with acetylation and methylation being extensively studied in relation to neurological disorders [172].

## 7.2.1 Histone acetylation in IS

The process of histone acetylation is governed by HATs (histone acetyltransferases), which add acetyl groups to lysine residues found in histones. This modification reduces the electrostatic interactions between histones and DNA, thereby enhancing the binding affinity of transcription factors and other proteins, which ultimately promotes gene expression. Conversely, HDACs(histone deacetylases) counteract this effect by compacting the chromatin architecture, resulting in transcriptional repression [173].

Experimental studies have indicated that ischemic stroke (IS) elevates the expression of HDACs in the brain, exhibiting spatial and temporal regulation. Decreases in histone acetylation during both the early and late phases after stroke correlate with aggravated injury and adverse outcomes [174-176]. Consequently, the majority of investigations into the role of histone deacetylation in IS have concentrated on the effects of pharmacological HDAC inhibition across diverse experimental models.

In rodent models of photothrombotic stroke, the SAHA (a broad-spectrum HDAC inhibitor suberoylanilide hydroxamic acid) and the MI192(a selective HDAC2/3 inhibitor) have been found to enhance the expression of neuroplasticity-related genes near the infarct, improve dendritic complexity and spine density, and decrease apoptosis within the infarcted region. Both treatments similarly led to reduced infarct volume and supported functional recovery following photothrombotic stroke [177, 178].

In rodent models of tMCAO, SAHA administration effectively reversed H3 deacetylation, enhanced the expression of anti-apoptotic proteins Bcl-2 and Hsp70, facilitated functional recovery, and lessened secondary brain injury following the event [176, 179]. Furthermore, the use of broad-spectrum HDAC inhibitors such as sodium butyrate or PBA (4-phenylbutyrate) has been shown to mitigate inflammation, edema, infarct size, apoptosis, and impairments in motor function in rodents after tMCAO [180-182].

In permanent MCAO models, TSA treatment resulted in decreased acetylation of histone H3, prevention of neurological deficits, and reduced cerebral injury [183]. Conversely, sodium butyrate administration attenuated microglial activation and promoted neurogenesis in the subventricular zone, dentate gyrus, striatum, and frontal cortex after permanent MCAO [183, 184]. Furthermore, the administration of class I HDAC inhibitors, including valproic acid (VPA), has exhibited neuroprotective properties in multiple stroke models [185-191]. Therefore, HDAC inhibition holds promise as a therapeutic strategy in various experimental stroke models and types of post-stroke.

## 7.2.2 Histone methylation in IS

In the context of histone methylation, lysine and arginine residues undergo modification by two classes of HMTs (histone methyltransferases): PRMTs (protein arginine methyltransferases) and KMTs (lysine methyltransferases). Histone lysine demethylases (KDMs) can demethylate histones that have been methylated by KMTs and PRMTs [192]. The specific histone type being modified and the position of the amino acid residue that serves as the substrate determine the influence of histone methylation on gene expression [172]. H3K4me or H3K48me (methylation of lysine 4 or lysine 48 on histone H3) is linked to enhanced gene expression, whereas H3K27me (methylation at lysine 27) is known to repress gene expression. Regulation of H3K4 represents one of the most thoroughly explored histone methylation processes in the context of IS.

In both the mouse internal carotid artery occlusion (ICAO) model and the rat photothrombotic stroke model, ischemic conditions can modulate the activity and expression of various HMTs and KDMs. In these models, an elevation in the levels of SUV39H1 and G9a is observed. Knockout or pharmacological inhibition of these enzymes in experimental models can enhance BDNF (brain-derived neurotrophic factor) expression, improve neuronal survival, and reduce apoptosis in ischemic injury zones as well as cortical infarct areas [155, 193, 194].

In rodent models of tMCAO, decreased activity of H3K4 histone methyltransferases has been observed in astrocytes of elderly female mice. In contrast, adult mice exhibit higher enrichment of H3K4me3 and H3K9me3, with increased expression of H3K4me3 on the VEGF gene [157]. H3K4me3 is associated with increased astrocytes in the cortical regions following tMCAO. Histone methyltransferases such as SET, which methylate H3K4 or H3K36, and protein containing the MYND domain 2 (SMYD2), show increased expression around cortical infarcts. Knockout of SMYD2 alleviates blood-brain barrier (BBB) disruption, edema, neurological impairments, and secondary cerebral damage after tMCAO in mice [195]. In vitro investigations using primary rat brain microvascular endothelial cells reveal that SMYD2 regulates the ubiquitin-dependent degradation of sphingosine kinase, essential for endothelial barrier integrity, via methylation following OGD. This suggests that SMYD2 exacerbates BBB disruption post-ischemia [195]. In transient global cerebral ischemia rat models, levels of LSD1, which induces transcriptional repression by removing H3K4 methylation, are elevated in the dentate gyrus, amygdala, and cortex from 1 hour to 3 days following reperfusion [196].

## 7.3 LncRNA in IS

**Figure 5.**
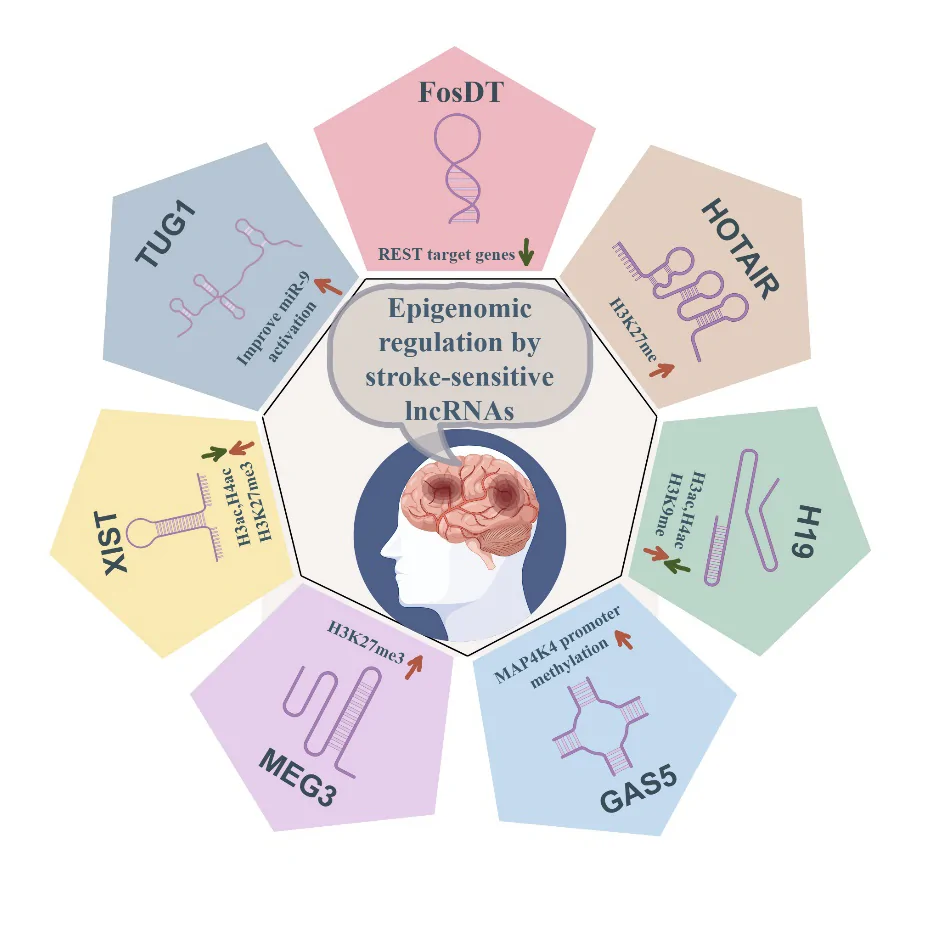
**Epigenomic regulation by stroke-sensitive lncRNAs**. LncRNAs engage with epigenetic regulators of DNA and histone modifications to precisely modulate the transcriptome following stroke. Key stroke-sensitive lncRNAs implicated in epigenetic regulation consist of FosDT, XIST, HOTAIR, MEG3, H19, GAS5, and TUG1.

Long non-coding RNAs (lncRNAs) refer to RNA transcripts exceeding 200 nucleotides in length that lack protein-coding capacity [197]. These lncRNAs are predominantly expressed in the brain and have been thoroughly investigated regarding their roles in neural development and neurological pathologies [198]. Studies have shown that hundreds of lncRNAs exhibit aberrant expression after stroke in both patients and experimental rodent models [199]. LncRNAs are being investigated as promising diagnostic and prognostic biomarkers for stroke [200], in addition to their roles as mediators of pathophysiological mechanisms in ischemic and hemorrhagic stroke rodent models [201, 202]. Previous investigations have identified key stroke-sensitive lncRNAs involved in epigenetic regulation, such as FosDT, HOTAIR, H19, GAS5, MEG3, XIST, and TUG1 (Fig. 5).

After tMCAO, REST inhibition has been demonstrated to relieve repression of genes associated with synaptic plasticity, decrease infarct volume, and enhance functional recovery in rats [203]. Research shows that several genes are promoted for epigenetic silencing by REST in CA1 hippocampal neurons, particularly after rats experience transient global ischemia [204]. The RE1 elements of target genes are bound by REST, and the core repressors Sin3A and coREST are recruited to mediate gene repression [205]. Fos downstream transcript (FosDT) is shown to interact with Sin3A and coREST, leading to the inhibition of REST target genes related to synaptic plasticity. The absence of FosDT results in the derepression of downstream genes regulated by REST, thus mitigating motor deficits and reducing infarct size [206, 207].

An additional lncRNA, HOTAIR (Hox transcript antisense intergenic RNA), displays prolonged expression patterns after permanent MCAO in murine models [208, 209]. Suppression of HOTAIR has been demonstrated to mitigate apoptosis, inflammation, and infarct size while enhancing motor and cognitive performance in mice post-permanent MCAO [209]. Following OGD, the expression of HOTAIR is markedly increased in the hBMVECs (human brain microvascular endothelial cells). Inhibition of HOTAIR leads to a reduction in cellular apoptosis and enhances the structural integrity of the hBMVEC layer by promoting the expression of tight junction proteins [210].

Evidence indicates that lncRNA H19 modulates chromatin alterations and is elevated in the rodent brain post-focal ischemia [211-214]. In mouse microglia exposed to oxygen-glucose deprivation (OGD), H19 promotes HDAC1 upregulation while concurrently reducing levels of acetylated histones H3 and H4 [215]. In a study involving rat neural stem cells subjected to OGD, H19 was shown to interact with chromatin remodeling proteins EZH2 and SUZ12, regulating the transcription of genes associated with hypoxia response, proliferation, and neurogenesis [214]. After tMCAO in rats, H19 exacerbates secondary brain injury by enhancing the expression of inflammatory mediators [216]. Inhibition of H19 results in elevated expression of IGF-1R, neurogenesis-associated proteins (including Notch1), and mTOR pathway genes, promoting axonal sprouting following tMCAO in mice [212, 217].

Furthermore, silencing H19 diminishes cerebral infarction and neurological impairments while enhancing performance in cortical-dependent motor tasks across various rodent models of focal ischemia [212-215, 217].

The long non-coding RNA known as GAS5 (growth arrest-specific transcript 5) has been demonstrated to facilitate the methylation of the promoter of MAP4K4 (mitogen-activated protein kinase kinase 4) via the recruitment of DNMT3B, which subsequently leads to diminished expression of MAP4K4 and a reduction in apoptosis within primary cortical neurons subjected to OGD [218]. Increased expression of GAS5 has been found to decrease infarction and improve cognitive recovery following tMCAO in mice by inhibiting MAP4K4 [218]. Conversely, silencing GAS5 reduces cell apoptosis and lowers cell survival by inactivating Notch1 in primary neuronal cultures exposed to OGD [219]. Clinical cohort studies have indicated that levels of GAS5 are elevated in the bloodstream of stroke patients, linking this elevation to an increased stroke risk. Additionally, high levels of GAS5 are linked to poorer recovery outcomes in patients with hypertension, diabetes, and elderly individuals [220, 221].

Long non-coding RNA MEG3 (maternally expressed gene 3) is upregulated in the core area following tMCAO in rodents but downregulated in the penumbra [222, 223]. It has been demonstrated that MEG3 contributes to the methylation of histone H3 by interacting with EZH2, the jumonji component of PRC2, and the AT-rich interaction domain 2 (JARID2) [224]. Furthermore, MEG3 interacts directly with the DNA-binding domain of p53, resulting in its activation and inducing apoptosis in ischemic models both in vitro and in vivo [223, 225, 226]. The silencing of MEG3 results in reduced levels of Bax and cleaved caspase-3 in hBMVECs, while simultaneously enhancing pro-angiogenic gene expression, including VEGF and HIF-1α, in rat BMVECs exposed to OGD [226]. Additionally, the knockdown of MEG3 also results in reduced infarct size and diminished neurological deficits after tMCAO [222, 227].

The long non-coding RNA known as X-inactive specific transcript (XIST) recruits repressive histone modifications, including HDAC3 and components of PRC2, to modulate gene expression [228]. After tMCAO, there was an increase in XIST expression, and the knockdown of XIST disrupted angiogenesis while exacerbating cerebral vascular injury in mouse models. In patients with ischemic stroke, XIST levels showed a decrease during the early stage (48 hours) but rose during the late stage (7 days) post-stroke; however, the overall levels of XIST were negatively correlated with the severity of neurological impairment [229].

The long non-coding RNA TUG1 is involved in the regulation of apoptosis in various cell types [230, 231]. Within the framework of ischemic stroke (IS), Chen et al. observed that TUG1 was overexpressed in the brains of rats subjected to MCAO. In this study, it was identified that TUG1 interacts with miR-9 in primary neurons exposed to oxygen-glucose deprivation (OGD), consequently diminishing its activity. miR-9, recognized as an anti-apoptotic factor, specifically targets the pro-apoptotic protein Bim-1. Suppression of TUG1 was shown to enhance neuronal survival during OGD by amplifying miR-9 activity [232].

Epigenetics, as a pivotal mechanism for regulating gene expression, is recognized as a significant marker and key factor in aging. Given that ischemic stroke is a prevalent form of acute brain injury, the extensive research in epigenetics highlights that our focus on IS research may need to expand beyond current perspectives. Gives us pause for thought that how such acute injuries might influence systemic aging over a prolonged lifespan. This alternative perspective could offer a novel approach to studying both IS and aging, enriching the research content and potentially uncovering new insights and breakthroughs in the future.

## 8. Loss of proteostasis and IS

Every cell employs a variety of quality control systems to preserve the stability and functionality of its proteome [233]. These systems operate in a synchronized manner to either correct the structure of misfolded peptides or entirely eliminate and degrade them, thereby preventing the build-up of damaged components and facilitating the ongoing renewal of cellular peptides [6]. Proteostasis represents a dynamic balance involving protein biosynthesis, folding, and degradation, facilitated through a complex network of cellular quality control mechanisms [234, 235].This network consists of mechanisms such as autophagy, SUMOylation(SUMO conjugation pathways), ubiquitination, and the unfolded protein response (UPR), all essential for cellular viability in the post-ischemic brain. In ischemic cerebral tissue, the delivery of critical oxygen and nutrients is inadequate to fulfill metabolic requirements. This results indisruption of calcium homeostasis, ATP depletion, and oxidative stress, which inevitably disrupts cellular proteostasis [236]. For instance, an overproduction of ROS negatively impacts the processes of protein degradation, alters protein structure, and disturbs the cellular and subcellular redox environments, leading to the accumulation of damaged and misfolded proteins in neural cells, which compromises proteostasis [237-239].

Although there is only indirect evidence suggesting that ubiquitination is involved in human brain ischemia [240], research in animal models has demonstrated an increase in ubiquitinated proteins within the brain after both global and focal cerebral ischemia [241-245]. Hochrainer and colleagues illustrated that ischemia-reperfusion results in a rise of ubiquitinated aggregates in mouse brains subsequent to transient arterial occlusion, with ubiquitination being more pronounced in the cortical regions surrounding the ischemic core compared to the striatum within the core of the ischemic damage [241]. In vitro experiments demonstrate that OGD triggers the ubiquitination of pro-apoptotic and postsynaptic proteins, enhancing their prompt degradation via the ubiquitin-proteasome pathway (UPS), which could play a significant role in the ischemic tolerance elicited by preconditioning [246, 247].

Serving as a protein quality control mechanism, SUMOylation has demonstrated neuroprotective effects in the majority of animal studies [248-252]. For instance, the overexpression of Ubc9 (a SUMOylation conjugating enzyme) in mice results in increased levels of SUMOylated proteins, providing neuroprotection against ischemia-induced cerebral injury [251]. The silencing of SUMO1-3 expression in the mouse brain, which inhibits SUMOylation, leads to inferior functional outcomes after tMCAO [253]. The knockdown of SUMO1 notably reduces the advantages of OGD preconditioning in cells [249]; additionally, SUMOylation levels rise following a short preconditioning duration in tMCAO [253]. Recent case reports have shown an elevation in SUMO1-3 immunoreactivity within neurons of the ischemic penumbra in human stroke, mirroring patterns found in animal studies [254].

Autophagy serves as an essential clearance pathway that is vital for preserving protein homeostasis. Prior research has shown that autophagy is induced in response to experimental cerebral ischemia [255-261]. Imaging analyses reveal a significant increase in neuronal autophagy activity in the peri-ischemic region of the mouse brain after IS [262]. Early investigations in animals indicated that the application of autophagy inhibitors, 3-methyladenine (3-MA) or bafilomycin A1, resulted in a substantial decrease in infarct volumes both prior to and following treatment [258, 263]. Nevertheless, a more recent investigation discovered that preconditioning utilizing 3-MA exacerbates stroke outcomes [264]. Moreover, the autophagy stimulant rapamycin, which inhibits mTOR signaling, has exhibited neuroprotective properties in cases of neonatal hypoxic-ischemic injury [255].

## 9. Disabled Macroautophagy and IS

The process known as macroautophagy, hereafter referred to as "autophagy," is characterized by the encapsulation of cytoplasmic substances within double-membrane vesicles called autophagosomes, which subsequently merge with lysosomes to facilitate the degradation of their internal contents [265]. Ischemia and hypoxia lead to cellular energy depletion, resulting in rapid cytoskeletal collapse and loss of ion homeostasis. An array of ischemic cascade reactions initiates apoptotic pathways [266]. Autophagy, recognized as a mode of cell death, is receiving increasing attention in the study of ischemic stroke.

## 9.1 Autophagy and neurons in IS

Studies investigating autophagy's involvement in ischemia-related neuronal damage have yielded differing conclusions. The predominant findings indicate that autophagy provides a protective mechanism against neuronal injury during IS. For instance, in rats subjected to an ischemia/reperfusion (I/R) model, autophagy plays a critical role in modulating endoplasmic reticulum stress after brain I/R [267], limiting the buildup of subcellular organelles and leading to organelle injury following transient brain I/R-induced autophagy [268]. Autophagy's neuroprotective effects during reperfusion might be linked to the removal of mitochondria and misfolded proteins [268], reduction of endoplasmic reticulum stress [269], and inhibition of apoptotic processes [270].However, other studies indicate that inhibiting autophagy can help prevent ischemia-induced neuronal damage [271-273]. The varied roles of autophagy in ischemia-induced neuronal injury suggest that it is not merely a straightforward cell death process. Different experimental models and levels of injury may trigger autophagy through different mechanisms, thus adapting to the needs of the organism at the time.

## 9.2 Autophagy and neuroglia and endothelial cells in IS

Autophagy is not only induced in neurons but also in glial cells. Microglia, the resident immune cells of the brain, become triggered in response to pathological stimuli [274]. In vitro hypoxia treatment of microglia has been shown to upregulate Hypoxia-inducible factor-1α and promote autophagic cell death in these cells [275]. Autophagy activation through the Akt/mTOR signaling pathway, regulated by reactive oxygen species (ROS), may facilitate apoptosis of microglia during ischemia/reperfusion injury [276]. These studies suggest that autophagy is activated in response to ischemia/hypoxia-induced microglial damage.

Astrocytes, which represent the predominant cell type within the central nervous system, serve as vital constituents of the blood-brain barrier and are essential for preserving normal brain function. Research indicates that both brain ischemia and oxygen-glucose deprivation trigger the activation of autophagy in astrocytes [277]. Administration of 3-MA, a known autophagy inhibitor, significantly mitigates astrocyte death induced by OGD [278]. Nevertheless, akin to neurons, conflicting studies suggest that autophagy might play a protective role in astrocytes subjected to ischemic stress, as the downregulation of this process results in reduced survival rates of astrocytes under OGD. Kasprowska et al. reported that following short-term OGD exposure, astrocytes treated with 3-MA displayed elevated levels of cleaved caspase-3 compared to controls, while prolonged OGD exposure yielded an opposing outcome [279].

Ischemic stroke followed by reperfusion can cause damage to the blood-brain barrier and enhance vascular permeability, which plays a role in the onset of cerebral edema and negative clinical outcomes in patients [280]. On one hand, the activation of the autophagy-lysosomal pathway after oxygen-glucose deprivation facilitates the breakdown of claudin-5, a crucial component of the BBB. Conversely, during the reperfusion phase, damage inflicted by ischemia/reperfusion (I/R) on brain microvascular endothelial cells results in BBB disruption, while upregulation of autophagy mediated by microRNA offers protection against OGD-induced injury in these cells [281]. Autophagy inhibition may protect brain microvascular endothelial cells from damage resulting from OGD/R [282]. In the initial stages of OGD/R caveolin-1 mediates the autophagy-lysosomal degradation of ZO-1 [283]. In conditions of ischemia, autophagy might play a role in endothelial injury and the disruption of the BBB.

## 10. Dysbiosis and IS

Current research has identified a link between dysbiosis and aging, with fecal microbiota transplantation experiments demonstrating the potential to reverse brain immunity and age-related cognitive impairments in aging brains [284-286]. Gastrointestinal complications resulting from stroke coexist [287-289] with negative outcomes following stroke discharge, including elevated mortality, deterioration of neurological function, and prolonged recovery [290-294]. Nonetheless, a relatively overlooked consequence of stroke involves gut microbiota dysbiosis, which not only heightens the risk of cerebrovascular incidents and plays a role in stroke occurrence but is also regarded as a contributor to systemic infections [295].

Studies have shown that the gut microbiota's impact on stroke outcomes is facilitated by bidirectional communication along the brain-gut axis. Investigations into focal cerebral ischemia in monkeys have demonstrated an elevated relative abundance of Prevotella, concurrently with a decline in the levels of Faecalibacterium, Streptococcus, Lactobacillus, and Oscillospira [296]. Lactobacillus, a crucial host beneficial probiotic, was found to be reduced in relative abundance after cerebral infarction in monkeys [296]. Supplementation with Lactobacillus has demonstrated benefits in enhancing cognitive function and mood, while also reducing age-related inflammation in experimental models [297-299]. The efficacy of Lactobacillus supplementation for post-stroke patients remains uncertain and warrants further exploration in upcoming clinical trials. Additionally, a reduction in the relative abundance of Streptococcus was observed following cerebral ischemia. The Streptococcus genus includes both probiotics (e.g., Streptococcus thermophilus) [300] and pathogenic bacteria (e.g., Streptococcus pneumoniae) [301]. These findings suggest that not only does stroke induce gut microbiota dysbiosis, but it also leads to chronic systemic inflammation. The growth of Bacteroidetes has been confirmed following cerebral ischemia in monkeys [296]. Likewise, a rise in the abundance of Bacteroidetes was noted in mice three days post-ischemic stroke, regarded as indicative of post-stroke dysbiosis [292]. Correlation studies additionally demonstrate a strong association between increased plasma lipopolysaccharide levels or inflammatory cytokines and the overgrowth of Bacteroidetes. Increased lipopolysaccharides in experimental monkeys' plasma, particularly at 6 and 12 months post-stroke, highlight the persistence of systemic inflammation following stroke [296]. Pro-inflammatory cytokines originating from the gut and entering the brain's culture layer can interact directly with the brain, worsening the pathological alterations that occur after a stroke [297]. As research continues, future studies on the mechanisms connecting stroke and aging will provide further insights into treatment strategies for both conditions.

## 11. Stem cell exhaustion and IS

Despite numerous studies on the use of neural stem cell therapies to mitigate IS [302-304], there is a lack of research on whether IS induces stem cell depletion. Although reports suggest increased neurogenic activity of neural stem cells (NSCs) and differentiated neurons in the subventricular zone following IS [305], excessive proliferation of stem cells and progenitor cells may potentially accelerate the depletion of the stem cell niche, leading to detrimental effects [306], such as impaired neuronal repair during subsequent injury post-stroke.

Various factors influencing the proliferation and differentiation of neural stem cells into functional neurons are associated with the pathophysiology of ischemic stroke. An example is BDNF, which is vital for preserving the NSC niche and promoting cellular differentiation [307]. Analyses of clinical data indicate that in the acute phase of stroke, the severity of the condition is inversely related to BDNF levels. Additionally, individuals experiencing acute stroke display markedly reduced serum BDNF levels when compared to healthy controls [308-311]. FGFs (fibroblast growth factors), a group of polypeptide growth factors, participate in numerous biological processes, such as neuronal survival and protection, while also facilitating neurogenesis and neuronal development in the brain. There are few clinical studies investigating the relationship between FGFs and stroke, with FGF-21 and FGF-23 being the most closely associated. Increased plasma concentrations of FGF-21 have been associated with unfavorable outcomes in patients experiencing acute ischemic stroke, indicating that FGF-21 could function as a prognostic biomarker for this condition [312]. FGF-23 is associated with microvascular-related brain damage, with higher FGF-23 concentrations correlating with increased risk of cardiogenic embolism, thereby indirectly suggesting that FGF-23 may be related to IS-induced damage [313].

## 12. Genomic instability and IS

Genomic instability arises from multiple factors, such as environmental stressors, DNA damage, telomere shortening, replication inaccuracies, and genetic mutations. The accumulation of genomic instability and damage escalates with aging, triggering DDR (DNA damage response) mechanisms aimed at repairing and averting additional DNA damage [314]. When DDR mechanisms are ineffective, several cellular outcomes may occur, including cell cycle arrest, apoptosis, and cellular senescence [315]. Within the framework of ischemic stroke (IS), genomic instability, particularly that resulting from telomere shortening, interplays with several other previously outlined Hallmarks. Changes in nuclear DNA, mitochondrial DNA, telomeres, DNA methylation, and various histone modifications during the IS damage process also drive alterations in genomic stability. Assessing factors influencing genomic stability may reflect age-related changes. For ischemic stroke (IS), the process of injury and the accumulation of damage can impact genomic stability. The chronic stimulation and destabilization following decompensation may gradually manifest over time.

## 13. Discussion

As noted by Carlos López-Otín et al. in their updated hallmarks of aging in 2023, the distinctions between the "hallmarks" are essentially dispersed, as they interact with each other and are not independent. Therefore, their classification is inevitably arbitrary. This aligns with the message I aimed to convey in Figure 1. In creating Figure 1, we sought a way to accurately express this concept, so we chose the Tai Chi diagram, rich in ancient Chinese philosophical meaning, to place at the center of the image, illustrating the 12 hallmarks and their relationship with ischemic stroke. As mentioned by Carlos López-Otín et al., the interdependence of aging markers implies that emphasizing or diminishing a particular hallmark in experiments often impacts other hallmarks as well. This emphasizes that aging is an intricate phenomenon that necessitates a holistic perspective.

As summarized in this review, the pathological processes of ischemic stroke are closely related to each hallmark of aging. Specifically, after the onset of ischemic stroke, brain tissue is susceptible to DNA damage due to hypoxia and nutrient deprivation, leading to genomic instability. During the repair of these damages, mutations may arise, affecting normal cellular function and promoting the aging process. Genomic instability can result in apoptosis or cellular senescence, which impairs subsequent repair capabilities. Epigenetic alterations induced by ischemia, such as DNA methylation and histone modifications, can influence gene expression—particularly genes associated with the cell cycle, metabolism, and inflammation—thereby accelerating cellular senescence and the loss of related functions. In this context, telomere damage and shortening occur in both neurons and glial cells, leading to reduced proliferative capacity and limiting the regenerative potential of brain tissue, exacerbating signs of aging. Cells exposed to oxidative stress and nutrient deprivation may experience protein misfolding and aggregation. The loss of proteostasis impacts fundamental cellular functions and can trigger chronic inflammation, further harming surrounding healthy cells. Moreover, mitochondrial dysfunction caused by ischemia decreases cellular energy production and increases oxidative stress. This mitochondrial impairment generates more free radicals, exacerbating cellular damage and aging. Simultaneously, autophagic function is compromised, preventing cells from clearing damaged organelles and proteins. The failure of autophagy leads to the accumulation of toxic substances within cells, accelerating cellular senescence and impairing regenerative capacity, making brain tissue repair increasingly difficult. As a tissue with extremely high nutritional demands, the brain is susceptible to metabolic dysregulation following ischemia, particularly through the abnormal activation of the mTOR pathway. This dysregulation leads to metabolic abnormalities and an imbalance in growth regulation, affecting cell survival while promoting cellular senescence and apoptosis. Ischemic stroke-induced cellular senescence is characterized by growth arrest, morphological changes, and the secretion of numerous pro-inflammatory cytokines. Moreover, it may impact surrounding healthy cells via the senescence-associated secretory phenotype (SASP), resulting in the formation of a "senescence-inflammation" cycle. Despite the lack of research on ischemic stroke and stem cell exhaustion, it is clear that stem cell depletion is closely associated with other hallmarks, such as cellular senescence and chronic inflammation, which can adversely affect the stem cell niche and hinder their differentiation and proliferation. Consequently, this limits the functional role of stem cells in the pathological processes of ischemic stroke. Following a stroke, cells suffer damage from DNA to organelles, naturally impairing their functions. Disruptions in intercellular communication can exacerbate immune responses, intensifying inflammation and hampering repair mechanisms, which negatively affects outcomes in ischemic stroke. In terms of the impact of ischemic stroke on peripheral organs, dysbiosis of the gut microbiota is closely linked to systemic inflammatory responses. This microbial imbalance may affect brain repair and function through the immune system, and its contribution to aging should not be overlooked. Chronic inflammation is a significant hallmark of aging and was separately discussed as one of the markers by Carlos López-Otín et al. in 2023. Chronic inflammation can be both a consequence of cellular senescence and an accelerant of the aging process. Importantly, it is an inevitable outcome of stroke or acute injury.

Overall, these 12 hallmarks can be broadly categorized into three tiers. The first tier, located at the base, represents the most microscopic markers: genomic instability, telomere attrition, and epigenetic alterations. The second tier moves beyond the genome to encompass proteins, organelles, and signaling pathways, including loss of proteostasis, mitochondrial dysfunction, impaired autophagy, and nutrient sensing dysregulation. The final tier, at the pinnacle of the pyramid, represents the most macroscopic markers related to cells, intercellular interactions, and microbiota: cellular senescence, stem cell exhaustion, altered intercellular communication, chronic inflammation, and dysbiosis.

It is evident that the relationship between ischemic stroke and aging is comprehensive and closely intertwined, manifesting "from the inside out". Regardless of the level involved, each aspect contributes to the overall progression toward aging. Upon reflection, this three-tiered progression mirrors the pathological processes of acute injury, from onset to resolution. This perspective helps clarify the purpose of our review: to emphasize the importance of understanding the relationship between acute injuries, particularly ischemic stroke, and aging. Acute injury should be considered an important factor in assessing aging and cannot be overlooked.

## 14. Future perspectives of IS and aging

Aging has traditionally been viewed as a change over time, closely associated with advancing age. Throughout history, humanity has been eager to explore and find ways to slow down aging and extend human lifespan. With the advent of an aging population, the rapid development of medicine has brought aging research to unprecedented heights. New studies continually suggest that certain interventions can achieve this goal, yet most research remains at the experimental animal stage. Human studies are scarce and largely focus on lifestyle changes such as diet, exercise, and sleep, which often yield slow and modest results. Researchers seem to concentrate on aspects with slower processes to uncover the secrets of aging. Stroke, the second leading cause of death globally, is particularly prevalent among elderly patients and has recently shown an increase in younger populations. When a stroke occurs, the primary focus is usually on managing the acute injury, aiming to improve prognosis, enhance quality of life, and reduce recurrence and disability. However, whether the acute event and its chronic damage accumulation also accelerate overall aging is often overlooked.

By summarizing various aspects of stroke and aging-related hallmarks, we can observe that the impact of stroke-induced damage on aging is pervasive. Although our synthesis and collection of individual aspects may not be exhaustive, the critical takeaway is that these studies indeed highlight that stroke significantly affects these biomarkers of aging. This underscores that stroke or acute injury should not be overlooked when assessing aging. As a research team in geriatrics and neurology, we have uncovered an often-neglected dimension of stroke's impact on aging while exploring stroke prevention and treatment strategies. With this review, we aim to elucidate the relationship between stroke, an acute injury, and aging-related hallmarks. By providing a comprehensive overview, we hope to offer researchers a broader perspective on how acute injury mechanisms intertwine with aging. We hope to present a new viewpoint and a more complete evaluation framework for future research and exploration in the field of aging.
